# Construction of Safety Early Warning Model for Construction of Engineering Based on Convolution Neural Network

**DOI:** 10.1155/2022/8937084

**Published:** 2022-09-16

**Authors:** Changge Zhao

**Affiliations:** Henan University of Engineering, Henan, Zhengzhou 45000, China

## Abstract

In recent years, China's engineering construction management level has been greatly improved, but compared with other industries, the construction industry still has low production efficiency, serious waste, and low level of information problems, and especially in the process of engineering management practice, schedule delay has become the focus of engineering management problems. With the continuous development of science and technology, computer and information technology have been continuously applied in engineering, among which deep neural network (DNN) technology, lean management, information visualization technology, and other technologies have become the hot spot of industry research, and the application of these emerging technologies to improve the level of project schedule control has become an urgent demand of the industry. Therefore, on the basis of deep learning, this paper analyzes the principle and application of object detection and feature extraction constructed by neural network and combines text feature extraction and image feature extraction methods. This application provides a new idea for the development of the construction industry.

## 1. Introduction

Cross-media unified expression is an important research direction in the multimedia field [1], which aims to connect the semantic gap between different modal data, such as images and texts, and establish a unified semantic expression. Based on the theory and method of cross-media unified expression, some popular branch directions are derived, for example, visual question answering, image caption, and visual grounding.

With the development and in-depth research of deep learning in recent years, many difficult problems in the field of computer, especially those related to computer vision and natural language processing, have made great breakthroughs. Even on some problems, algorithms based on deep learning have surpassed human capabilities. Due to the powerful representation capability of DNN [2], deep learning algorithms have become the mainstream research direction in the field of cross-media.

Cross-media target retrieval has a wide range of application scenarios [3, 4]. Text or voice with dialogue question answering system has been widely used in mobile terminals and PC operating systems, as an important way of human-computer interaction, for example, Siri of Apple, Cortana of Microsoft, and Alexa of Amazon. Some wearable smart devices enable human-computer interaction, such as Microsoft HoloLens. In the future, text-based image object retrieval systems may become an important way of human-computer interaction, changing people's traditional production and lifestyle. This technology can greatly improve the efficiency of text-based image retrieval and object retrieval in the image.

At present, DNN technology in domestic architecture is still in the initial stage of exploration, and the theoretical technology and experimental research are still lagging behind the market demand for engineering practice. Therefore, we need to combine DNN technology software and project management methods in foreign buildings with China's national conditions to find out the applicable technology methods and standards suitable for China's construction industry. In the practice of architectural design and engineering management, how to improve the efficiency of architectural design, effectively control the schedule, dynamically and rationally allocate all kinds of resources needed, and improve the scientific, efficient, and accurate management of architectural design and construction project are the problems to be solved.

In the field of construction engineering, many researchers have used deep learning to implement helmet detection. For example, the use of the convolutional neural network (CNN) to automatically extract image features for helmet detection is a topic that is still in the exploratory stage, and the technology is not yet mature. In the helmet detection process, computer vision is used to automatically detect whether a worker at a construction site is wearing a helmet, including body detection, helmet detection, and matching. When a mismatch is detected between the spatial position of the human body and the helmet, a safety alert is issued [4, 5].

**Figure 1:**
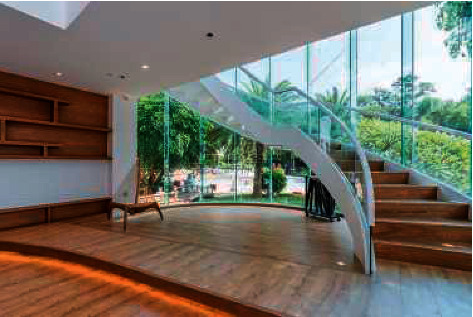
Indoor space layout sampling environment.

In addition, the interior spatial layout is a typical application of deep learning in construction engineering [6]. With the development of 3D visual information processing technology, it is important to apply it to architectural interior space layout in combination with digital image processing technology in interior space planning and design. A safety alert is issued; visual parameter feature analysis methods are used to construct a building interior space layout feature extraction analysis model to achieve building interior space layout detection, thereby improving the quality of building interior space layout planning [7]. For the optimisation scheme of building interior space, deep learning can effectively extract building interior space features, identify image edge pixels, and determine the visual image points of the building interior space layout. A visual feature parameter fusion model for interior spatial layout is established as shown in Figure 1.

## 2. Related Work

### 2.1. Cross-Media Retrieval

Due to the basic characteristics of different media data, there is a problem of heterogeneity gap [8]. Many early researchers used traditional feature analysis methods such as principal component analysis and partial least square method. The subspace of the same dimension is obtained by dimensionality reduction of the underlying features of different media data. However, the subspace obtained by dimensionality reduction will lose some underlying features with potential correlation information, so it is difficult to accurately reflect the relationship between them in high-level semantics. Pay attention to the correlation of different media data contents, and use canonical correlation analysis [9] to find the correlation of different media data in the underlying feature space. At the same time, subspace mapping is carried out on this basis, which not only solves the heterogeneity of the underlying features but also ensures their correlation to the maximum extent.

**Figure 2:**
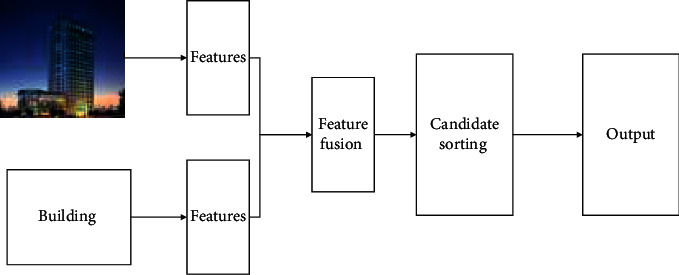
Cross-media retrieval and processing of construction engineering data.

In the topic of buildings, the building retrieval algorithm can be abstracted into an algorithm model containing three modules, for example, the method of building image location by reconstructing query phrases [10], the media retrieval method based on MCB [11], and the method of retrieving architectural image targets according to natural language [12, 13]. The general steps can be divided into three steps [14]. The first step is to generate candidate boxes and feature extraction. Given an input architectural image, a candidate box generation model is used to generate many candidate boxes and the corresponding visual features of each candidate box. DNN model is used to encode query text to extract text features. The second part is multimodal feature fusion. The visual features and query text features of each candidate box are fused using a cross-modal feature fusion algorithm. The simplest fusion methods, such as splicing and point multiplication, are used to complete cross-modal feature fusion. The third step is target positioning. A target box prediction model is designed to predict and output the most relevant target coordinate box of the query text on the image using the fusion feature obtained in the previous step. When processing building images or building texts, the general structure of cross-media retrieval is shown in Figure 2.

### 2.2. Application of DNN in Construction Engineering

Deep learning has a history of more than half a century. It first appeared in the early 1940s. From 1943 to now, deep learning has experienced three stages of development. The first is the beginning stage of deep learning, which is marked by the appearance of the MCP model, which lays the foundation for deep learning. The second is the rising stage of deep learning. Some researchers have put forward the concepts of BP neural network and activation function, making the neural network that has fallen into silence gradually become the focus of attention. The third stage, that is, the stage that deep learning is currently experiencing, is the outbreak of deep learning. The word “deep learning” has officially come into people's vision, and various deep neural network models shine in recognition tasks. Among them, the restricted Boltzmann machine, deep belief networks (DBN) [15], and CNN are excellent models in the field of deep learning.

In 2006, Hinton et al. proposed the concept of deep learning. Thus, in-depth learning has stepped into the third stage of development. In the 2012 ImageNet image recognition competition, Hinton's research group established the AlexNet model to reduce the error rate of ImageNet image classification to 15%. Taigman et al. proposed DeepFace model and achieved the accuracy of 97.35 in LFW face database [16]. In addition, the aldrop regularization method has achieved good results on image data sets. The development of deep learning has entered the fast lane.

**Figure 3:**
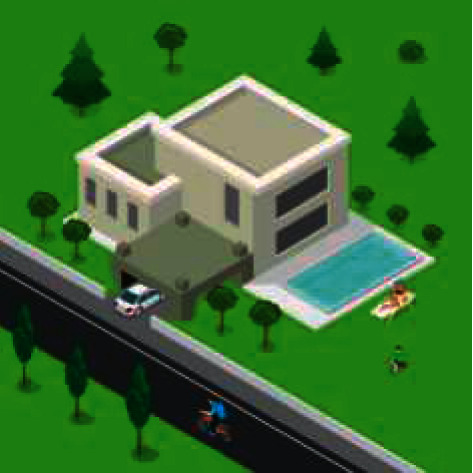
Animation 3D building visualization.

In recent years, with the development of deep neural networks, various models have emerged CNN and recurrent neural networks, and other models perform well. CNN is one of the best models in deep learning. R-CNN can be used to improve the efficiency and visualization of 3d modeling. Mask R-CNN algorithm is used to segment architectural images, build 3D visualization models, and generate modeling images as shown in Figure 3.

Smart city has gradually become a new term in current urban architecture. The research focus in the process of smart city construction is to quickly realize the common three-dimensional visualization of indoor buildings and reduce the modeling cost. Many researchers have tried this. For example, 3D laser scanning combined with point cloud data is used for 3D modeling. Using the RCB-D indoor scene 3D space modeling giant [17, 18], the depth learning method is introduced into the RCB-D indoor modeling to effectively improve the flexibility and accuracy of indoor modeling.

### 2.3. Three-Dimensional Model Data

The 3D model is a rich and complex collection of information in a single point, line, and surface. In order to eliminate the effects of interference, conflicts, and noise, we need to preprocess the 3D model data to find the essential features of the model and minimise the extraction of nonessential features. In principle, the more key features are extracted from a model of the same species, the more accurate the model will be in terms of recognition and retrieval. Models have fixed shape features in the human subjective consciousness at different coordinates; however, for machine processing, they are very different. Therefore, the different processing of different coordinates and the subsequent retrieval of the model have a huge impact. The effectiveness of model preprocessing directly determines, in a simple and clear way, the accuracy of generalisation, classification, identification, filtering, and retrieval of models.

**Figure 4:**
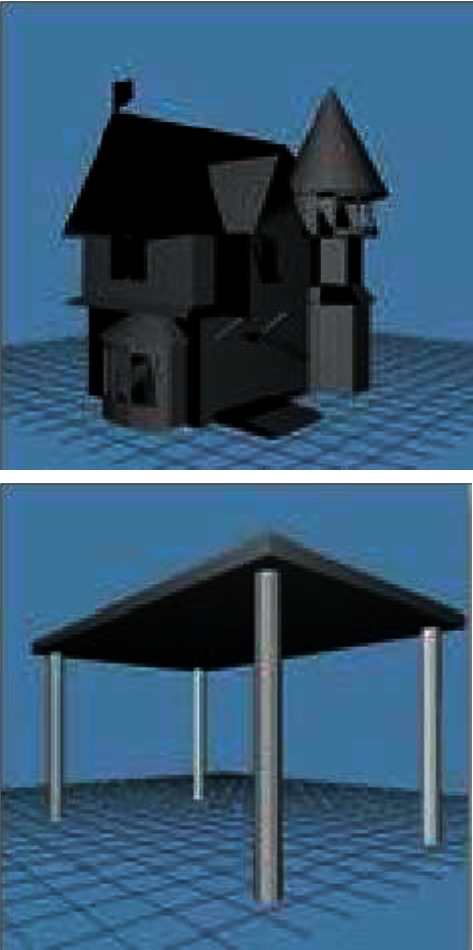
3D model of a building.

The experiments conducted in this paper are based on model data from the Princeton University shared 3D model standard library. This standard library contains several categories, and each category also contains a sufficient number of typical sample models, which were analysed, compared, identified, and selected by researchers from over 6000 sample models to form the Princeton University Library (PSB Library). The PSB contains 1814 sample models, and the two architectural 3D models in the library are shown in Figure 4.

The purpose of the 3D model standard library is to provide a reference evaluation system for clustering, classification, identification, and retrieval of 3D models. This benchmark library (PSB), for each 3D model, contains a detailed file (of the type off format with polygons) with a text file containing the original data information and a file with a jpg image containing a thumbnail of the 3D model. The 3D model standard library is a standardised and uniform format for normalising 3D models into off format (a very common polygon format at the University of Minnesota Centre for Advanced Mathematical Geometry). When the 3D model is converted uniformly, the scene information, textures, and color information about the 3D model is eliminated and simply stored as a set of vertices and polygons in a surface index set, with the core of the process being the geometric surface information. Saving the file in off format is very simple, the first line is prominently marked “OFF,” and the second line clearly indicates the number of vertices, facets, and edges. The number of edges can usually be ignored automatically. The vertices are listed in *x*, *y*, and *z* coordinates, one per row. The facepiece information follows the list of vertices (the number of vertices is specified), which is distributed one per row, and finally the index list of vertices.

### 2.4. Deep Neural Networks

The important idea of DNN is that it has more “depth” than the common neural network models, i.e. a deep neural network contains multiple hidden layers, rather than just “input layer-hidden layer-output layer.” The main types of deep neural networks are deep belief network and restrict Boltzmann machine and convolutional neural network. In this paper, we learn a new DNN by modifying its parameters and apply it to traffic sign recognition to observe the effect of different network structures on the recognition effect.

CNN is a new type of network based on the unique structure of a neuron that can reduce the complexity of feedback neural networks identified by researchers when observing the cat's brain cortex. CNNs are widely used in the field of pattern classification because they can avoid explicit feature extraction from images and can be trained directly on the original input image.

The network structure of a DNN alternates between multiple convolutional and subsampling layers, with each layer of neurons connected only to the nodes of the previous layer. As a multilayer feature extractor, the input to the DNN is the original pixel intensity of the image, which is extracted by the convolutional and subsampling layers to form a feature vector, which is then fully connected to the output neuron for classification.

## 3. Cross Media Building Detection Based on DNN

### 3.1. Core Ideas for Architectural Images in DNNs

At present, there are different problems with light, occlusion, and deformation when capturing images of buildings in realistic environments. Different preprocessing methods have their own advantages in highlighting different features or removing certain disturbances. In order to effectively avoid errors in the acquisition, effectively utilise the advantages of different preprocessing methods, and improve the recognition effect of the algorithm, it is proposed to use different preprocessing methods on the data set, form different training sets, overcome the disturbing information in the samples from different angles, and finally make a comprehensive evaluation to make the training results more accurate. At the same time, different structures of deep neural networks (different number of feature maps or different sampling methods) are built on the same preprocessed dataset to achieve fast recognition of distorted building images and to ensure the efficiency and correctness of the algorithm.

Before the start of each training session, the training set is preprocessed and input to different deep neural networks, thus forming the multicolumn deep neural network MCDNN. The MCDNN combines the DNNs trained on different samples linearly and then performs fuzzy judgement on the output results to improve the recognition effect.

### 3.2. Cross-Media Building Detection Based on DNN

For building image processing tasks in cross-media detection, VGGNet and RseNet [19] have excellent feature expression ability, so they are often used to encode visual features of images. VGGNet is an in-depth CNN developed by the computer vision group of Oxford University. VGGNet explored the relationship between the depth of CNN and its performance and built a deep CNN by repeatedly stacking small convolution cores and pooling layers. In addition, the official website of VGGNet has open-source models trained on ImageNet datasets. Researchers can easily use VGGNet in their own research fields and retrain it. Therefore, VGGNet is often studied to extract the visual features of images, making it almost a standard for image feature extraction.

**Figure 5:**
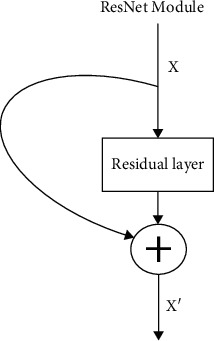
ResNet.

ResNet (Figure 5) is a deep neural network model proposed by Professor He[20]. One of the main problems solved by ResNet is the degradation of deep neural networks. When the network depth increases, the performance of the network becomes saturated or even decreases. However, the neural network with more upper layers can extract more complex feature patterns, that is, the deeper the network, the better the theoretical performance. He et al.proposed an identity mapping operation [21], which uses direct connection to connect the front and back layers of the network. In this way, the gradient vanishing problem in the network is well solved, and the number of layers of the deep neural network can be greatly increased.

The birth of CNN was initially influenced by the visual cognitive mechanism of biology. In 1962, in the experiment of studying the visual cortex cells of cats, it was found that each visual neuron only processes a small piece of visual image, which is called the receptive field. In 1984, the concept of neocognitron was proposed, and the first CNN model was born.

Although CNN is a neural network improved on the feedforward neural network, it is different from the traditional feedforward neural network in that the neurons between its adjacent layers are not fully connected. A complete CNN structure usually consists of five parts. The first is the input layer, which can directly use the original image as input and then repeatedly perform convolution and pooling operations on multiple convolution layers and pooling layers to abstract the information in the image into features with higher information content and then enter the full connection layer to synthesize the previously extracted features. Finally, with the calculation of softmax, the probability classification is output to complete the classification task.

### 3.3. Application of Cross-Media Object Detection in Buildings

In various media, construction engineering data are usually displayed in the form of pictures or text. Taking the sample of text construction engineering as an example, its essence is transformed into natural language processing. Query text in natural language is usually of variable length. In order to facilitate subsequent modeling and processing, it is usually necessary to convert the text into a fixed length vector in cross-media target retrieval methods. This method of converting natural language text into digital vectors is also called text feature coding. Traditional text feature coding models include one hot model [22], vector space model [23], and word2vec model [24, 25].

**Figure 6:**
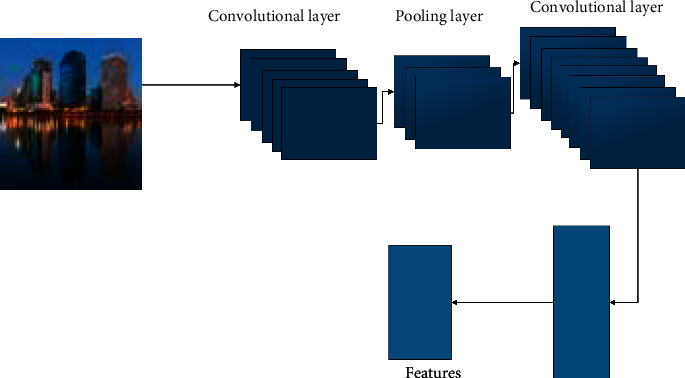
Object detection building with CNN.

For the image samples of construction projects, with the maturity of the neural network, there are many image detection methods that can be selected. In general, AlexNet, VGg, and CNN are the mainstream algorithms. In 2012, Hinton et al. A 8-layer AlexNet won the championship in 2012. Compared with LeNet-5, it has a similar overall structure and deeper network layers. Since then, researchers have continuously put forward new improvement and improvement methods, and new CNN structures emerge in endlessly. It includes VGGNet from layer 16 to 19, GoogleNet from layer 22, and RESNET from layer 152. From these CNN structures, we can see that the remarkable feature of the development of the CNN model is that the number of layers is getting deeper and the structure is becoming more complex. The building target recognition based on CNN is shown in Figure 6.

## 4. Feature Extraction of Building Engineering Based on DNN

### 4.1. Building Text Feature Extraction

Since the emergence of human civilization, the text has existed in people's daily production and life. As one of the oldest information media, text still has an irreplaceable position in modern life. TF-IDF, LDA, and other models are commonly used for feature extraction of text.

TF-IDF refers to word frequency–reverse file frequency, where term frequency (TF) is word frequency and inverse document frequency (IDF) indicates the inverse text frequency index [26]. TF-IDF is a way to vectorize features and then extract them. It can evaluate the importance of a word or phrase in a document set or corpus. That is, if a word appears more in a document than in other documents, it means that the word can be used as a classification basis to distinguish the document from other documents.

Latent Dirichlet allocation (LDA) [27] is a document topic probability generation model, LDA can detect the potential topic probability distribution in the corpus. It is also a three-level Bayesian probability model. The three structural levels are document, topic, and word.

### 4.2. Cross Media Image Feature Extraction

As an intuitive and visible media type, image is the most direct source for people to obtain information from the outside world through their eyes. For the local areas and details of the image, the global feature cannot show its superiority, so we also need to study the local features of the image.

SIFT is a local feature descriptor for images proposed by David Lowers in 1999 [28]. The working principle of SIFT algorithm is to search for some key feature points distributed in the target image. These feature points are “special” in the whole image. They can also remain stable when the brightness of the image changes and the rotation angle and scale change significantly [29]. In short, searching the key feature points of images in different scale spaces is the core of the working principle of SIFT algorithm.

### 4.3. Application of Cross-Media Feature Extraction in Construction Engineering

The image features of buildings captured by human eyes in natural conditions are mainly area, shape, color, and spatial relationship. We see the height of the building, the floor area, and the texture of the building surface. These features describe the construction project from the overall relationship of the building, so it has the characteristics of intuitive performance and good invariance. Color histogram statistical algorithm, SIFT, and Gabor texture feature extraction algorithm are commonly used, which can realize global feature extraction algorithm. Among them, SIFT is a mature algorithm that can extract local features.

When the construction engineering data and samples are documents, the TF-IDF algorithm is used for processing. Suppose there is a construction engineering corpus *D* and a topic set *t*, document set *D* contains multiple documents *D*, topic set *t* contains multiple topic *T*, and a single document *D* also contains multiple topic *t*. The TF-IDF model uses topics as the middle layer of words and documents, rather than directly dealing with the relationship between words and documents, which can more accurately reflect the semantic information of documents.

When the construction samples are images, SIFT algorithm is used to extract features. That is to search the key points in the building image, such as segmentation line, unique shape, and appearance, these feature points are unique in the whole building. Therefore, it can remain stable when the brightness of the building image changes, and the rotation angle and scale change significantly. In other words, the key to SIFT feature extraction is to search the key feature points of building structures in different scale spaces.

## 5. Conclusion

Based on the background of construction engineering and the demand for cross-media, this paper introduces the application principle of multiclass deep network in cross-media detection from the perspective of deep learning, including VGg algorithm, ResNet, and CNN. When extracting building structure and building features, if the sample is a document, TF-IDF or LDA algorithm can be given priority. If the building sample is an image, CNN has the best processing effect. This paper has made a certain summary and application description on how to effectively identify and extract construction engineering, which has reference significance. In the future, we will collect enough building samples for verification experiments to deeply analyze the advantages and disadvantages of each depth neural network.

## Data Availability

The dataset used in this paper is available from the corresponding author upon request.

## References

[1] Qi J., Peng Y., Yuan Y. (2018). Cross-media Multi-Level Alignment with Relation Attention Network.

[2] Yu H., Tao J., Qin C. (2022). A novel constrained dense convolutional autoencoder and DNN-based semi-supervised method for shield machine tunnel geological formation recognition. *Mechanical Systems and Signal Processing*.

[3] Vidgen B., Yasseri T. (2020). Detecting weak and strong Islamophobic hate speech on social media. *Journal of Information Technology & Politics*.

[4] Li A., Du J., Kou F. (2022). Scientific and Technological Information Oriented Semantics-Adversarial and Media-adversarial Cross-media Retrieval.

[5] Wei C. H., Allot A., Leaman R., Lu Z. (2019). PubTator central: automated concept annotation for biomedical full text articles. *Nucleic Acids Research*.

[6] Latif A., Rasheed A., Sajid U. (2019). Content-based image retrieval and feature extraction: a comprehensive review. *Mathematical Problems in Engineering*.

[7] Oncescu A. M., Koepke A., Henriques J. F., Akata Z., Albanie S. (2021). Audio Retrieval with Natural Language Queries.

[8] Zhou X., Zhang Y., Guo H., Jiang B. (2021). Towards bridging the structure gap in heterogeneous catalysis: the impact of defects in dissociative chemisorption of methane on Ir surfaces. *Physical Chemistry Chemical Physics*.

[9] Haldorai A., Ramu A. (2021). Canonical correlation analysis based hyper basis feedforward neural network classification for urban sustainability. *Neural Processing Letters*.

[10] Shuai W., Li J. (2022). Few-shot learning with collateral location coding and single-key global spatial attention for medical image classification. *Electronics*.

[11] Agapaki E., Brilakis I. (2022). Geometric Digital Twinning of Industrial Facilities: Retrieval of Industrial Shapes.

[12] Bai S., Zheng Z., Wang X. Connecting language and vision for natural language-based vehicle retrieval.

[13] Feng Q., Ablavsky V., Sclaroff S. (2021). Cityflow-nl: Tracking and Retrieval of Vehicles at City Scale by Natural Language Descriptions.

[14] Xiang C. (2019). Cross media target retrieval based on deep learning.

[15] Javeed M., Gochoo M., Jalal A., Kim K. (2021). HF-SPHR: hybrid features for sustainable physical healthcare pattern recognition using deep belief networks. *Sustainability*.

[16] Wang M., Deng W. (2021). Deep face recognition: a survey. *Neurocomputing*.

[17] Tan H., Liu X., Yin B., Li X. (2022). Cross-modal semantic matching generative adversarial networks for text-to-image synthesis. *IEEE Transactions on Multimedia*.

[18] Prasetyo E., Suciati N., Fatichah C. (2021). Multi-level Residual Network VGG for Fish Species Classification Journal of King Saud University-Computer and Information Sciences.

[19] Wightman R., Touvron H., Jégou H. (2021). ResNet Strikes Back: An Improved Training Procedure in Timm. https://arxiv.org/abs/2110.00476.

[20] He K., Zhang X., Ren S., Sun J. Deep residual learning for image recognition.

[21] He K., Zhang X., Ren S., Sun J. Identity mappings in deep residual networks.

[22] Buckman J., Roy A., Raffel C., Goodfellow I. Thermometer encoding: one hot way to resist adversarial examples.

[23] Salton G., Wong A., Yang C. S. (1975). A vector space model for automatic indexing. *Communications of the ACM*.

[24] Jang B., Kim I., Kim J. W. (2019). Word2vec convolutional neural networks for classification of news articles and tweets. *PLoS One*.

[25] Fukushima K. (1988). Neocognitron: a hierarchical neural network capable of visual pattern recognition. *Neural Networks*.

[26] Ramos J. (2003, December). Using TF-IDF to determine word relevance in document queries. *Proceedings of the first instructional conference on machine learning*.

[27] Blei D. M., Ng A. Y., Jordan M. I. (2003). Latent dirichlet allocation. *Journal of Machine Learning Research*.

[28] Gupta S., Thakur K., Kumar M. (2021). 2D-human face recognition using SIFT and SURF descriptors of face’s feature regions. *The Visual Computer*.

[29] Bay H., Tuytelaars T., Gool L. V. Surf: speeded up robust features.

